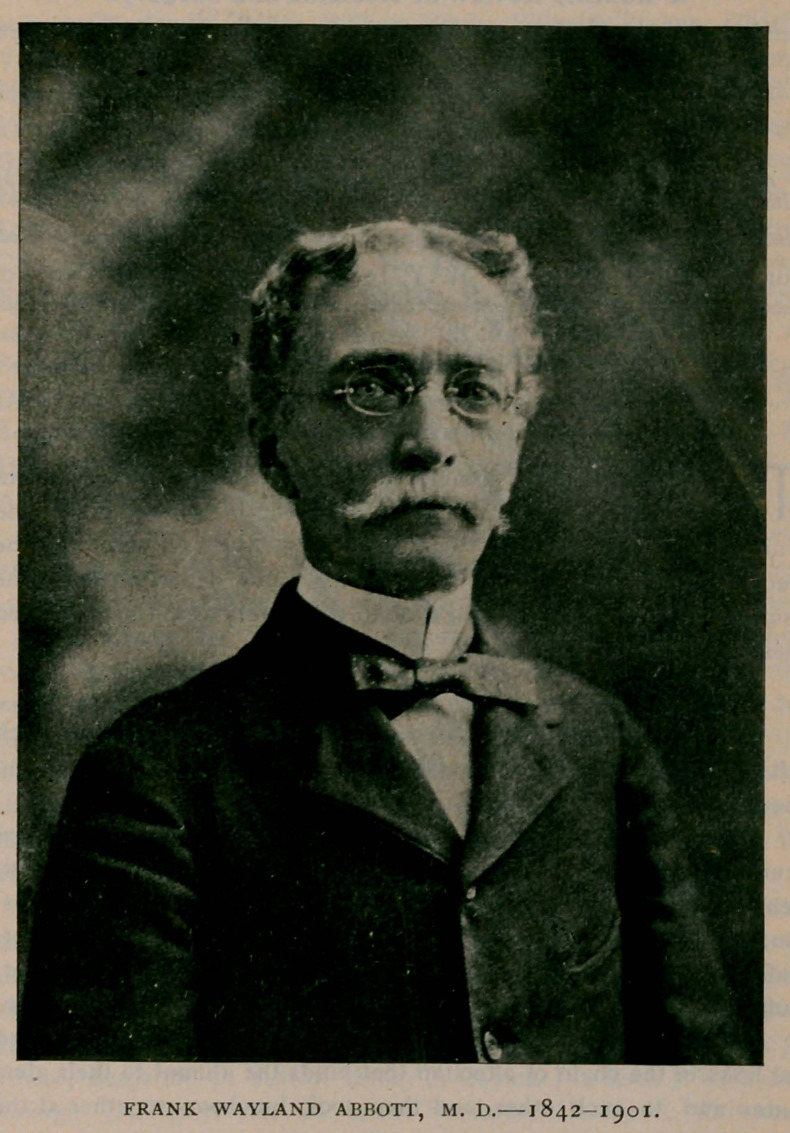# Medical Society of the County of Erie

**Published:** 1901-05

**Authors:** Franklin C. Gram


### MEDICAL SOCIETY OF THE COUNTY OF ERIE.

### MEMORIAL MEETING.

REPORTED BY FRANKLIN C. GRAM, M. D., Secretary.

A LARGE number of medical practitioners from Buffalo and vicinity, attended the special meeting of the Medical Society of the County of Erie, held in the Young Men's Christian Association parlors, April 11, 1901, at 5 p.m., in memory of Dr. Frank W. Abbott, an esteemed member of the profession, who died April 9, 1901, aged 59 years.

The president, Dr. W. C. PHELPS, in opening the meeting, briefly alluded to the honorable career of the deceased, who graduated in the same class with the president in 1866, and requested the society to take suitable action. He also stated that several other medical organisations had been invited to participate in this meeting.

On motion of Dr. COAKLEY the president appointed Drs. Howe, Rochester and Thos. Lothrop, a committee on memorial.

The committee retired and later presented the following:

### MEMORIAL.

As we, the members of the Medical Society of the County of Erie, have learned, with deep regret, of the death of our associate, Dr. Frank Wayland Abbott, we desire to place on record an expression of our appreciation of the great esteem in which he was held.

During the many years that he has worked with us, we have learned to admire his sterling qualities of mind and heart, his strict professional integrity, and the attributes which made him an honor to his profession and a representative of what was best and most admirable as a citizen.

We appreciate especially his services rendered to the medical profession of this city, his careful attention to details of his work, his patience in dealing with difficult questions, and, above all, the high ideals of justice and of professional honor which have marked his course throughout.

In recognition of his admirable character we unite in an expression of deepest regret at the great loss sustained by this society, in sympathy for his family, and for all who came within the circle of his kindly and beneficent influence.

LUCIEN HOWE,
DE LANCEY ROCHESTER,
THOMAS LOTHROP.

Dr. Grove said : It is a painful duty that I, in common with the rest of you assembled, am asked to perform today. I have known Dr. Abbott for twenty-three years. While a student at the medical college and interne at the Buffalo General Hospital, I first became acquainted with him, but I learned to know him more intimately as my colleague at the Charity Eye, Ear, Nose and Throat Hospital. He was one of the founders of this hospital ten years ago. I express only the sentiments of its directors and attending staff when I say it was with pride that he labored for its advancement. Dr. Abbott was always a gentleman in the high and lofty sense, and of strong religious conviction. Speaking of him professionally, he was conservative and reliable. He would discommode himself to favor another. He could not tolerate the methods employed by the trimmer. He would not make use of unbecoming methods in seeking place and position. You could rely upon him for he was candid, frank, and honest. The profession is benefited along the line of true ethics by the life and example of such a man. We shall all miss him and still delight to remember that we knew one endowed with such ennobling qualities.

A telegram was received from Dr. Frederick F. Hoyer, of Tonawanda, in which he expressed his great sorrow at the death of his friend.

The great esteem in which Dr. Abbott was held then manifested itself in the beautiful eulogies which were rendered by many of those present. Dr. James W. Putnam had known him for many years and spoke of his scholastic abilities and his many kindnesses to patients and to young practitioners.

Dr. Irving M. Snow gave a brief account of his last illness. Brief, but feeling remarks were also made by Drs. Howe, Starr, Rochester, Wyckoff, Coakley, Lewis, Pettit, Jewett, Hubbell and James S. Smith.

The memorial was then adopted and the meeting adjourned.

---

### ACTION OF THE PENSION SURGEONS.

The Buffalo Board of U. S. Pension Examining Surgeons, has heard with sorrow of the demise of our associate Dr. Frank W. Abbott, and offer the following brief tribute to his memory.

#### IN MEMORIAM.

#### FRANK WAYLAND ABBOTT, M. D.—1842–1901.

We are impressed most deeply at the great loss the service has sustained through the death of Dr. Abbott, when we recall the judicial fairness of all his work relating to the examinations of claimants for pension. His professional skill, always of a high order, was often tested in the extreme, yet never found unequal to the occasion, and the final decision always commanded the approbation of his associates as well as of the Pension Bureau at Washington.

To ourselves the loss is a personal one, for we had each not only come to respect his professional attainments, but to regard him as a friend. His motives were so pure, his ideals so high, and his per-

FRANK WAYLAND ABBOTT, M. D.—1842–1901.

sonality so charming, that we feel as though our little family had been entered and one of its most essential members taken.

We extend to the surviving members of Dr. Abbott's family our deepest, tenderest sympathy.

William Warren Potter,
Justin G. Thompson.

## Figures and Tables

**Figure f1:**